# A population-based survey on tanning bed use in Germany

**DOI:** 10.1186/1471-5945-9-6

**Published:** 2009-07-20

**Authors:** Franziska U Börner, Holger Schütz, Peter Wiedemann

**Affiliations:** 1 Institute of Neurosciences and Medicine, INM-8: Ethics in the Neurosciences, Forschungszentrum Jülich, 52425 Jülich, Germany

## Abstract

**Background:**

The suntanning industry has grown up over the last decade in Europe, mainly because tanned skin is considered socially desirable and attractive. Because of the potential negative impact of artificial tanning on public health, this study was to investigate tanning bed use behaviour, UV related risk perception and beliefs about tanning in the German population.

**Methods:**

In 2007, a representative telephone survey was carried out among 1501 German residents aged 14 years and older.

**Results:**

More than one fourth (28%) of the German population have used tanning beds at least once before in their lifetime. High-frequency tanning behaviour, i.e. using tanning beds more than 10 times per year, were recorded for 11%. Men and women aged 18 to 44 years and young women under the age of 18 used tanning beds more frequently (>10 times per year). Tanning bed use was positively related to appearance and lifestyle related beliefs as well as to the perception that tanned skin is healthy.

**Conclusion:**

This analysis indicates that tanning bed use is common in Germany. The positive relationships of appearance and health related beliefs with tanning bed use are of great concern. The results indicate underlying misconceptions about the positive effect of artificial UV radiation compared to natural UV radiation particular for high-frequency tanners. The data shows the importance as well as the limitations for risk communication in its current effort to inform effectively about the dangers of artificial UV radiation.

## Background

The prevalence of indoor tanning and the use of commercial tanning facilities has rapidly increased and gained widespread popularity over the past decade. Among most frequent reasons suggested for this development are the public's perception that artificial tanning is safe or even healthy [1,2], preparation of the skin before sun exposure [3] and the desire to tan for appearance reasons [4,5]. However, long term exposure to ultraviolet (UV) radiation is known to cause skin cancer, photoaging, cataracts, and immunosuppression [6]. Moreover, several epidemiological studies reported a relationship between deliberate exposure to artificial UV radiation (sunbeds/sunlamps) and cutaneous malignant melanoma [3,7-11]. Despite numerous warnings from public health organizations and medical experts [12], previous studies found that many people are not aware or are ignoring the detrimental effects of exposure to artificial UV light [2,13,14]. Of particular concern is the widely practiced and increasing popularity of tanning bed use by minors [15-20] which led several countries to establish legal requirements for the use of tanning beds by minors [21]. At present, German legislation does not restrict the use of tanning beds, but strict regulations that will prohibit their use by minors are under discussion [22]. However, research suggests that such legal restrictions may have only limited effect on tanning bed use by minors. For the US, Cokkinides et al. (2009) report no change in the prevalence of indoor tanning by minors from 1998 to 2004 [2], although an increasing number of states have restricted their use for this group.

In light of these developments and the ongoing efforts of several German public health agencies to involve operators of tanning salons voluntarily in following established safety standards and providing consumer consultations regarding UV health risks, the objectives of the present paper were (1) to investigate the frequency of tanning beds use in Germany, (2) to characterize tanning bed users, and (3) to assess attitudinal factors for tanning bed use among Germans to provide baseline figures for future public health campaigns regarding tanning bed use. As other studies and reviews have tried to investigate the psychological motives behind tanning bed use before [23], we attempt to explain tanning bed use behaviour within the German population by using variables from the established Health Action Process Approach model developed by Schwarzer [24,25].

## Methods

In May to June 2007, a national representative telephone survey was carried out among German residents aged 14 years and older. The survey was sponsored by the German Federal Office for Radiation Protection (BfS) to provide comprehensive baseline estimates of sun exposure, sun protection behaviour, artificial tanning behaviour, sun related knowledge and information seeking behaviour regarding UV information among the German population.

For this type of research no formal ethical approval procedure is necessary in Germany, however ethical approval of the survey questionnaire was obtained from the sponsoring body, the German Federal Office for Radiation Protection (BfS), to ensure good quality standards.

The telephone survey was conducted by a professional survey company, using a random digit dial procedure to access households and then selecting the respondent according to the so-called "last birthday" method, that is, selecting that household member age 14 or over who has had the last birthday. More than 11,000 households were approached to achieve the desired sample size of 1501 persons. The overall response rate was 13 percent, whereby the largest proportion of nonresponses was due to a general refusal to participate in a survey (62%). Another 25 percent refused because of disinterest in the specific topic of the survey, which was mentioned at the introduction as dealing with sun protection on behalf of the Federal Office for Radiation Protection (*Bundesamt für Strahlenschutz*). Despite this low response rate the survey sample does not differ substantially from the German population with regard to the distribution of demographic characteristics such as sex and age (see Table 1). There are, however, differences with regard to level of education: lower levels of education are underrepresented and higher levels overrepresented in this survey. Implications of the low response rate for the interpretation of results will be considered in the discussion section below.

**Table 1:** Comparison of the distribution of socio demographic characteristics of the telephone survey with the official statistics for Germany

	Survey %	Survey weighted %	Official statistic* %
**Sex**			
male	46.5	48.4	48.3
female	53.5	51.6	51.7
**Age Group**			
14 – 19	6.5	7.4	8.0
20 – 29	8.5	12.6	12.8
30 – 39	15.5	18.1	17.6
40 – 49	22.5	18.6	17.7
50 – 59	18.1	14.5	14.5
60 – 69	16.4	14.5	15.6
70 – 79	9.9	11.0	9.5
80 – 89	2.6	3.2	3.7
90 and above	0.1	0.1	0.6
**Education**			
Still in education Ausbildung	2.8	3.6	5.6
< High School	23.7	24.8	41.3
= High School Diploma	34.5	32.1	24.8
> High School	38.4	38.5	19.9

* Source: [26]

As this study focuses on tanning behaviour and its determinants, variables related to exposure habits to artificial tanning by frequency and time spent in each session, tanning related beliefs, risk perception, as well as beliefs about UV related adverse health effects and demographic characteristics are investigated, which were all part of the general sun behaviour and sun protection questionnaire used in the survey.

In order to explore potential determinants of tanning bad use, binary logistic regression was used. Two dichotomous dependent variables were created based on the two tanning bed use questions (general tanning bed use: coded as 1 for Tanning bed use and 0 for non-use; frequent tanning bed use: 1 for high-frequent use and 0 for low-frequent use). The criterion for high-frequency use was more than 10 tanning bed sessions per year and for low-frequency use 10 or less tanning bed session per year according to international safety recommendations from the World Health Organization [6]. Further, to facilitate data analysis and interpretation, dichotomous independent variables were created for each of the explaining health behaviour and risk variables by using median split. The data were analyzed with the Statistical Package for the Social Sciences (SPSS 15). As for all other analyses, data were weighted to take into account the age, sex and education distribution of the population, on the basis of the most recent German census [26]. The following scales were included as explanatory variables: (1) *Hazardousness UV *as summed index across judgements of severity and probability of five UV related adverse effects (cataract, sunburn, skin cancer, hair loss, skin aging), each assessed on a 10-point scale; (2) *Perceived personal risk *(10 point rating scale), and three tanning and sun related judgements with (3) *Tanned skin is attractive *(5 point rating scale), (4) *Tanned skin is healthy *(5 point rating scale) and (5) *Sun feels good *(5 point rating scale).

## Results

The final sample of 1501 respondents represents a weighted total of the German population with 52% females and 48% males. The majority of respondents had completed high school and beyond (74%) and were married 47%. The respondents ranged in age from 14 to 90 years, with an average age of 48 years (Table 2).

**Table 2:** General population characteristics and comparisons of tanning bed user and non-user characteristics

	**Sample** (n = 1501)		Tanning Bed users* (n = 426)		**Non-user** (n = 1075)		Sig.
Variable	N	%	N	%	N	%	p
Sex							
Male	727	48.4	166	22.8	561	77.2	.000
Female	774	51.6	260	33.6	514	66.4	
Age group							
14 – 17	81	5.4	15	18.5	66	81.5	.000
18 – 29	220	14.6	86	39.1	134	60.9	
30 – 44	426	28.4	184	43.2	242	56.8	
45 – 59	341	22.7	75	22.0	266	78.0	
60+	432	28.8	64	14.8	368	85.2	
Level of education							
< High School Diploma	373	26.0	83	22.3	290	77.7	.004
= High school Diploma	483	33.7	148	30.6	335	69.4	
> High School Diploma	578	40.3	182	31.5	396	68.5	
Marital status							
single	561	37.4	173	30.8	388	69.2	.001
married	697	46.5	204	29.3	493	70.7	
widowed	139	9.3	19	13.7	120	86.3	
divorced	103	6.8	28	27.2	75	72.8	

*All respondents who reported to have used tanning beds at least once before in their lifetime and more.

### General Tanning Bed Use

Overall, more than one fourth (28%) of the participants have used tanning beds at least once in their lifetime. More females than males have used tanning facilities (34% vs. 23%). Tanning bed users are significantly more often female, slightly younger as compared to non-users (40 vs. 49 years) and possess more often a high school diploma or higher. Among the overall population, the 30 to 44 years old respondents are the biggest group to have used tanning beds before (43%), followed by younger adults (18 to 29 years) with 39% and 45 to 59 year olds with 22%. Adolescents (<18 years) and respondents aged 60 and older have used tanning beds slightly less (19% and 15%). Comparisons of characteristics among tanning bed users and non-users are shown in Table 2.

In an attempt to explain general tanning bed use, health behaviour change variables from Schwarzer's HAPA model were used [24]. As shown in Table 3, results from the binary logistic regression analysis indicate that, the belief that "tanning is attractive" was significantly associated with tanning bed use (p < .001), with respondents who felt that tanning was attractive being 75% more likely to use tanning beds than respondents who didn't feel that way. In addition, respondents' judgements of severity and probability of UV related adverse health effects were found to be marginally related to tanning bed use(p07)None of the other explanatory variables showed any significant association. The explanatory power of the logistic regression model is with a Nagelkerke's Pseudo-R^2 ^of 0.022, however, low.

**Table 3:** Impact of explanatory variables on tanning bed use (general & frequent)

	**General tanning bed use** (n = 1078)*			**Frequent tanning bed use** (n = 318)**		
Variable/determinants	OR	95% CI	p	OR	95% CI	p
Hazardousness UV***	1.283	.982 – 1.678	<.07	.960	.593 – 1.555	.869
Perceived Personal Risk***	1.000	.758 – 1.318	.999	.989	.605 – 1.618	.965
„Tanning is attractive“	1.732	1.297 – 2.313	<.001	1.398	.830 – 2.355	.207
„Tanned skin is healthy“	.856	.650 – 1.128	.270	2.387	1.489 – 3.826	<.001
„Sun feels good“	.933	.712 – 1.223	.615	1.885	1.170 – 3.038	<.01

*Binary logistic regression with respect to general tanning bed use. Odds ratio with 95% confidence intervals. Tanning bed use dichotomized: users (1)/non-users (0). (User n = 317; non-user n = 761, Nagelkerke's Pseudo-R^2 ^= 0.022)

**Binary logistic regression with respect to frequent tanning bed use. Odds ratio with 95% confidence intervals. Tanning bed use dichotomized: high-frequent users (1) vs. low-frequent users (0). (High-frequent Users n = 138; low-frequent user (< 10 sessions per year & tanning bed users who had not tanned in the last 12 months) n = 180, Nagelkerke's Pseudo-R^2 ^= .107)

*** As defined in the Methods Section.

### Frequent Tanning Bed Use

61% of the tanners (n = 257) can be characterized as low-frequent users, i.e. people who tanned less than 10 times during the last year or people who indicated to have used tanning beds before in their life but had not done so in the last 12 months. 39 Percent (n = 169) of tanning bed users, on the contrary, reported to have used tanning beds more than 10 times during the previous 12 months.

**Figure 1:**
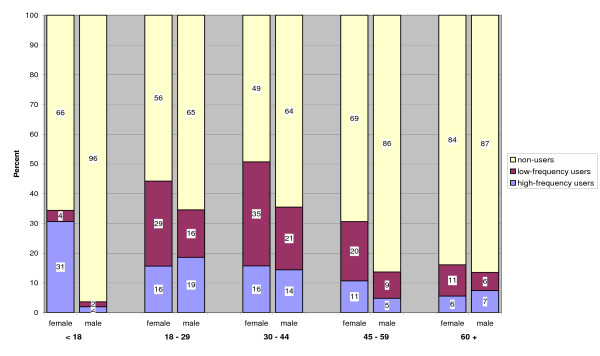
**Comparison of tanning bed use by age & gender**.

Slightly more female than male respondents used tanning beds more frequently (56% vs. 44%). Figure 1 exhibits significant gender related difference in tanning bed use for three of the five age groups.

For the under 18, 30 to 44 and 46 to 59 year olds, statistically significant differences were found in tanning bed use between men and women. For the 33 to 44 year olds the differences result from differences between the low frequency female and male users (35 vs. 21%) (p < .05). For the two other groups the proportion of both high- and low frequency users markedly differs for men and women. This is most noticeable in the adolescent group, there the proportion of young women under 18 who are high-frequency users of tanning beds is significantly higher compared to young men under 18 (p < .01). Only 2% of young males are high-frequent tanners compared to 31% of young females. Women aged 45 to 59 not only tend to tan more frequently compared to men in the same age group (11 vs. 5%) but are also more low-frequent tanners (20 vs. 9%) (p < .001). In the age groups 18 to 29 and 60+ no statistically significant differences between females and males in their tanning bed use could be found.

Interesting, however, is the fact that male respondents aged 18 to 44 years old show similar high-frequent tanning behaviour as women in the same age groups. The frequency of tanning bed use did not vary according to family status or level of education (see Figure 1). However, on average high-frequency tanning bed users tanned significantly longer compared to low-frequency tanners (13 vs. 10 minutes/session, p < .001). Table 4 shows that high-frequency users tanned more in the critical time range of 15 to 20+ minutes compared to low-frequency tanners (44% vs. 27%) who preferred to spend less time (<5 to 14 minutes) during each tanning session (56% vs. 73%) (Table 4).

**Table 4:** Time spent per tanning session by tanning bed use

Tanning Bed Users*	Total (%)	< 5 min. (%)	5 – 9 min. (%)	10 – 14 min. (%)	15 to 19 min. (%)	20+ min. (%)
Users	426	39 (9)	85 (20)	158 (37)	80 (19)	64 (15)
>10 session Users	169	7 (4)	25 (15)	62 (37)	35 (21)	40 (24)
=/< 10 session Users	257	32 (12)	59 (23)	96 (37)	45 (18)	25 (10)

* Tanning bed users: users = all respondents who reported to have at least used tanning beds once before in their life (n = 426); > 10 sessions = all respondents who reported to have used tanning beds more than 10 times in the last 12 months; =/<10 times in the last 12 months or at least once in their life before.

Looking again at potential determinants for frequency of tanning, results of the binary logistic analysis indicate that "tanned skin is healthy" was significantly associated with frequent tanning bed use, with tanners who felt that tanned skin was healthy being more likely to tan more than 10 times a year compared to those who didn't feel that way (p < .01). "Sun feels good", the strongest predictor for frequent tanning bed use next to "tanned skin is healthy", increased the likelihood of tanning bed use among high frequent tanners at more than 80% (p < .01). The explanatory power of the logistic regression model is with a Nagelkerke's Pseudo-R^2 ^of 0.228 within a normal range (Table 3).

## Discussion

While older studies from the late 80 s and early 90 s often report rather low rates of tanning bed use, more recent studies [4,14,27] find similar rates as in the present survey.

Although a substantial number of German respondents indicated to have used tanning beds at least once before in their life, a smaller proportion uses them on a more frequent basis. In our study, 11% of the sample have visited tanning salons more frequently (>10 times/year) whereas 17% of all respondents could be described as low-frequent tanners who either used tanning beds 10 times and less in the past year or had used tanning beds before but not at all in the last year. Other studies have reported similar frequencies of use [5,13,14]. Further noticeable is that high-frequency tanners in Germany spent more time in each tanning session compared to low-frequency tanners.

Previous research from the USA and Sweden shows that indoor tanning use varies between girls and boys (ranging from 12% to 37% in girls and 2% to 11% in boys) [15,21,20,28]. Our data shows similar trends for young adolescents. The frequent use of artificial tanning devices was found to be particular common for young female users (14 to 18 years old), supporting similar data from the USA [15,16,29]. The data underlines the alarming tendencies of young girls' indoor tanning behaviour and adds to the discussion about legislation to limit minors' access to commercial tanning facilities [2,21].

Although indoor tanning behaviour of adolescents and young adults are regularly investigated [2,15-20], only sparse data is available concerning adult tanning bed users' characteristics and tanning bed use frequency [4,27,30]. Our results showed an interesting age-related pattern for tanning bed use. While young women (<18 years old) use tanning beds more frequently than men in the same age group, in later age groups up to the age of 44 the difference between women and men in frequent tanning is diminishing. Men seem to start tanning later in life, a conclusion that is also supported by data from Mathys and colleagues indicating higher percentages of the 30+ age group using tanning beds [4]. Contrary to other studies [e.g. [4,9,14,30]] tanning bed users more often had a high school diploma (or higher) than non-users. This particular result might be due to the underrepresentation of participants with less than high school diploma in the present survey, and particularly those who were tanning bed users. With regard to frequent tanning bed use, no associations were found with demographic variables.

Of particular interest in our study is that for explaining general tanning bed use, attractiveness was the only prevalent motivation from all analyzed motivating factors. The data indicates that respondents who felt more strongly that tanned skin was attractive, were also more likely to have used tanning beds at least once before. This finding is similar to other studies, suggesting that that appearance related motivations are strong factors for tanning bed use [5,17,31,32]. For instance, in Switzerland, 92% of the respondents indicated that "appearance" was the major motivation to use tanning beds [4]. However, the low explanatory power of our model suggests that there might be other relevant determinants that we did not address in our survey. Candidates are peer group pressure and parental role model [2,33] that have been found to contribute to tanning bed use.

Looking at tanners, it is noticeable that the high-frequent user group were further motivated by the feeling that tanned skin is healthy and that sun feels good compared to respondents who tanned less frequently. These findings are similar to reasons reported elsewhere such as using tanning beds to protect the skin before going on vacation [14], to relax, to treat skin disease (acne, eczema), and general health improvement [13,32,34,35]. Other reports show that a high proportion of frequent tanning bed users believe that obtaining a tan from an artificial device would protect them from adverse health effects of sun exposure or that tanning beds are safer than the sun [13,32]. We believe that high-frequency tanning bed users in our German study may also attribute tanned and healthy skin to artificial UV sources. It seems possible that the popularity of indoor tanning facilities is due to the common belief that indoor tanning produces a safer tan than one caused by natural sun exposure [36,37]. Respondents may have felt that artificial tanning could protect them from the dangers of natural UV-radiation. Several studies found that operators of tanning salons contributed to the dissemination of false or misleading information about the consequences from the use of tanning devices [38-40].

A limitation of the present study is its low response rate of 13 percent. Although the problem of low response rates is by no means unique to this study (see, e.g. [41], for an overview), it certainly poses the questions whether this may introduce a bias and whether the results of the present study can be considered valid and, as intended, representative for the German population. Low response rates per se are, however, no reason for concern. There is no logical connection between a low response rate and nonresponse bias [42], and in fact empirical studies have shown that low response rate do not necessarily yield biased results. For instance, Keeter and colleagues [43], comparing the results of two surveys using identical questionnaires with response rates of 36% and 61%, found an average difference of about 2 percentage points across 91 comparisons. No difference exceeded 9 percentage points and most of the statistically significant differences were among demographic items. As noted above, the demographic structure of the present survey does not substantially differ from the German population (with one exception). But this does not imply, of course, that there are no differences with regard to other parameters which are of interest in this study, viz. tanning behaviour and its psychological determinants. Previous research suggests that the salience of the survey topic for the potential participants' lives influences their willingness to participate (see [44,45] for a discussion of this and related issues). Not surprisingly, people who are interested in the study topic are more likely to participate in the survey than those who are not. In the present study, 20 percent of the nonresponses are due disinterest in the topic of the survey: sun protection.

Although we have no information how disinterest in the topic sun protection is connected with actual tanning behaviour it seems plausible to assume that those who are disinterested will not care much about potential health risks and thus will not show a health-conscious tanning behaviour. Of course, it can also be that the reason for being disinterested in sun protection is that one is simply not interested in tanning and is not using tanning beds. In any way, this suggests that if there is a response bias in the present study, it will likely be in the direction of underestimating the frequency of tanning bed use in general and it might also be responsible for the inverse relationship between tanning bed use and level of education that runs counter to the results of other studies. However, it will not much affect the sex and age differences in this regard as well as the psychological potential determinants of tanning behaviour.

## Conclusion

In conclusion, this study indicates that tanning bed use is rather common in the German population, particularly among the 30 to 44 year olds for both genders and very young women under the age of 18.

Our data suggest that appearance based and lifestyle oriented motives are important motivating factors for tanning bed use, both for whether one decides to use tanning beds at all and for frequency of tanning. The results raise the question whether informing and educating the population primarily about skin cancer risks and dangers associated with tanning bed use is the right topic to be addressed if one wants to foster responsible tanning bed use. In fact, Knight and colleagues [46] have found that awareness of health risks due sun lamp use does not affect tanning behaviour. Rather, to effectively decrease the rate of artificial tanning, it seems that public perception and peer group opinion will have to change regarding what is aesthetically admirable. Here, public health information and education efforts have to address more prominently appearance and life-style related motives for tanning. Nevertheless, it will take a joint effort from health care organizations, media and physicians to bring about an eventual change in the belief that tanned skin is attractive or healthy.

However, the results also suggest that targeted risk information seems particularly necessary for high-frequency tanners. Here, future risk information should be set on possible misconceptions regarding artificial UV radiation sources and their individual harming or, as indicated in our data, perceived positive health effect. Public information campaigns, physicians, and particular dermatologists' interaction with patients will need to emphasize more about the adverse health effects associated with tanning bed use such as photo aging and cataracts. Particular educational focus should be given to the noticeable negative appearance based health outcome as well as addressing some of the existing misconceptions regarding artificial UV radiation.

## Competing interests

The authors declare that they have no competing interests.

## Authors' contributions

FUB participated in the design of the study, the development of the questionnaire, performed the statistical analysis and drafted the manuscript and wrote the paper. HS participated in the design of the study, the development of the questionnaire, performed the statistical analysis and revised the manuscript. PW conceived and supervised the study, participated in the design, the development of the questionnaire and writing the paper. All authors read and approved the final manuscript.

## Pre-publication history

The pre-publication history for this paper can be accessed here:



## References

[B1] Karagas M, Stannard VA, Mott LA, Slattery MJ, Spencer SK, Weinstock MA (2002). Use of tanning devices and risk of basal cell and squamous cell skin cancers. J Nat Cancer Inst.

[B2] Cokkinides V, Weinstock M, Lazovich D, Ward E, Thun M (2009). Indoor tanning use among adolescents in the US, 1998 to 2004. Cancer.

[B3] Autier P, Dore JF, Lejeune F, Koelmel KF, Geffeler O, Hille P, Cesarini JP, Lienard D, Liabeuf A, Joarlette M, Chemaly P, Hakim C, Koeln A, Kleeberg UR (1994). Cutaneous malignant melanoma and exposure to sunlamps and sunbeds: An EORTC multicenter case-control study in Belgium, France and Germany. Int J Cancer.

[B4] Mathys P, Moser M, Bressoud D, Gerber B (2002). Benützungsverhalten von Solarienbesucherinnen und -besuchern in der Schweiz. Soz-Präventivmed.

[B5] Bruggers JH, de Jong WD, Bosnjakovic BF, Passchier WF, Passchier WF, Bosnajakovic BF (1987). Use of artifical tanning equipment in the Netherlands. Human exposure to ultraviolet radiation – risks and regulations.

[B6] World Health Organization (2005). Protection against exposure to ultraviolet radiation, Geneva: WHO/EHC/UNEP. http://www.who.int/mediacentre/factsheets/fs287/en/index.html.

[B7] Swerdlow AJ, English JS, MacKie RM, O'Doherty CJ, Hunter JA, Clark J, Hole DJ (1988). Fluorescent lights, ultraviolet lamps, and risk of cutaneous melanoma. BMJ.

[B8] Walters SD, Marret LD, From L, Hertzman C, Shannon HS, Roy P (1990). The association of cutaneous malignant melanoma with the use of sunbeds and sunlamps. Am J Epidemiol.

[B9] Westerdahl J, Olsson H, Masbäck A, Ingvar C, Jonson N (1994). Use of sunbeds or sunlamps and malignant melanoma in southern Sweden. Am J Epidemiol.

[B10] Westerdahl J, Ingvar C, Masbäck A, Jonsson N, Olsson H (2000). Risk of cutaneous malignant melanoma in relation to use of sunbeds: further. Br J Cancer.

[B11] The International Agency for Research on Cancer Working Group (2006). The Association of Use of Sunbeds with Cutaneous Malignant Melanoma and Other Skin Cancers: A Systematic Review. International Journal of Cancer.

[B12] Johnson KR, Heilig LF, Hester JF, Francis SO, Deakyne SJ, Dellavalle RP (2006). Indoor tanning attitudes and practices of US dermatologists compared with other medical specialists. Arch Dermatol.

[B13] Mawn VB, Fleischer AB (1993). A Survey of attitudes, beliefs, and behavior regarding tanning bed use, sunbathing, and sunscreen use. J Am Acad Dermatol.

[B14] Rhainds M, De Guire L, Claveau J (1999). A population-based survey on the use of artificial tanning devices in the Province of Quebec, Canada. J Am Acad Dermatol.

[B15] Geller AC, Colditz G, Oliveria S, Emmons K, Jorgensen C, Aweh G (2002). Use of sunscreen, sunburning rates, and tanning bed use among more than 10,000 US children and adolescents. Pediatrics.

[B16] Balk SJ, Geller AC (2008). Teenagers and Artificial Tanning. Pediatrics.

[B17] Lazovich D, Sweeney C, Forster J (2005). Prevalence of Indoor Tanning Use in Minnesota, 2002. Arch Dermatol.

[B18] Boldeman C, Jannson B, Dal H, Ullen H (2003). Sunbed use among Swedish adolescents in the 1990s: a decline with an unchanged relationship to health risk behaviours. Scand J Public Health.

[B19] Haas AF (2007). Teens and Tans: Implementing Behavioral Change. Arch Dermatol.

[B20] Demko CA, Borawski EA, Debanne SM, Cooper KD, Stange KC (2003). Use of Indoor Tanning Facilities by White Adolescents in the United States. Arch Pediatr Adolesc Med.

[B21] Lazovich D, Forster J (2005). Indoor tanning by adolescents: prevalence, practices and policies. Eur J Cancer.

[B22] Bundesministerium für Umwelt und Gesundheit (2009). http://www.bmu.de/pressemitteilungen/aktuelle_pressemitteilungen/pm/40005.php.

[B23] Arthey S, Clarke VA (1995). Suntanning and sun protection: a review of the psychological literature. Soc Sci Med.

[B24] Schwarzer R, Schwarzer R (1992). Self-Efficacy in the Adoption and Maintenance of Health Behaviors: Theoretical Approaches and a New Model. Self-efficacy: Thought control of action.

[B25] Renner B, Schwarzer R, Suls J, Wallston KA (2003). Social-cognitive factors in health behavior change. Social psychological foundations of health and illness.

[B26] Demographische Standards 2004. Statistisches Bundesamt, Wiesbaden. http://www.gesis.org/dienstleistungen/tools-standards/standarddemographie/.

[B27] Ezzedine K, Malvy D, Maugner E, Nageotte O, Galan P, Hercberg S, Guinot C (2008). Artificial and natural ultraviolet radiation exposure: beliefs and behaviour of 7200 French Adults. JEADV.

[B28] Brandberg Y, Ulen H, Sjoberg L, Holm LE (1998). Sunbathing and sunbed related use related to self-image in a randomized sample of Swedish adolescents. Eur J Cancer Prev.

[B29] Robinson JK, Kim J, Rosenbaum S, Ortiz S (2008). Indoor Tanning Knowledge, Attitudes, and Behavior Among Young Adults From 1988–2007. Arch Dermatol.

[B30] Bränström R, Ullen H, Brandberg Y (2004). Attitudes, subjective norms and perception of behavioural control as predictors of sun-related behaviour in Swedish adults. Prev Med.

[B31] Danoff-Burg S, Mosher CE (2006). Predictors of tanning salon use: behavioral alternatives for enhancing appearance, relaxing and socializing. J Health Psychol.

[B32] Lillquist PP, Baptiste MA, Witzigman MA, Nasca PC (1994). A population-based survey of sun lamp and tanning parlor use in New York State. J Am Acad Dermatol.

[B33] Hoerster KD, Mayer JA, Woodruff SI, Malcarne V, Roesch SC, Capp E (2007). The Influence of Parents and Peers on Adolescent Indoor Tanning Behavior: Findings from a Multi-City Sample. J Am Acad Dermatol.

[B34] Diffey BL (1986). Analysis of risk of skin cancer from sunlight and solaria in subjects living in northern Europe. Photodermatol.

[B35] Hillhouse J, Turrisi R, Shields AL (2007). Patterns of Indoor Tanning Use: Implications for Clinical Interventions. Arch Dermatol.

[B36] Randle HW (1997). Suntanning: differences in perceptions throughout history. Mayo Clin Proc.

[B37] Sayre RM, Dewdy JC (2003). Sunbathing vs. indoor tanning: a realistic perspective. Photodermatol Photoimmunol Photomed.

[B38] Fairchild AL, Gemson DH (1992). Safety information provided to customers of New York City suntanning salons. Am J Prev Med.

[B39] Hester EJ, Johnson KR, Crane LA, Schilling LM, Dellavalle RP (2004). Indoor UV tanning operator opinion regarding youth access: An electronic survey. J AM Acad Dermatol.

[B40] Freeman SR, Francis SO, Lundahl K, Bowland T, Dellavalle RP (2006). UV tanning advertisements in high school newspapers. Arch Dermatol.

[B41] Groves RM, Peytcheva E (2008). The impact of nonresponse rates on nonresponse bias – A meta-analysis. Public Opinion Quarterly.

[B42] Stang A (2003). Nonresponse research – an underdeveloped field in epidemiology. Eur J Epidemiol.

[B43] Keeter S, Miller C, Kohut A, Groves RM, Presser S (2000). Consequences of reducing nonresponse in a national telephone survey. Public Opinion Quarterly.

[B44] Galea S, Tracy M (2007). Participation rates in epidemiologic studies. Annals of Epidemiology.

[B45] Groves RM, Presser S, Dipko S (2004). The role of topic interest in survey participation decisions. Public Opinion Quarterly.

[B46] Knight JM, Kirincich AN, Farmer ER, Hood AF (2002). Awareness of the risks of tanning lamps does not influence behaviour among college students. Arch Dermatol.

